# Preliminary studies on dynamics of *Culicoides *spp. In western Romania in conjunction with some environmental factors

**DOI:** 10.1186/1756-3305-7-S1-O7

**Published:** 2014-04-01

**Authors:** EM Tilibaşa, G Dărăbuş

**Affiliations:** 1 Department of Parasitology and Parasitological Diseases, Faculty of Veterinary Medicine,Banat's University of Agricultural Sciences and Veterinary Medicine "King Michael I" Timisoara, Romania

## 

*Culicoides *spp. play an important role as vectors for bluetongue and their dynamics is markedly influenced by the environmental changes. Based on these considerations, a study was conducted during May-June 2013 in the west area of Romania, which aimed capturing and identifying *Culicoides *spp. and tracking their population dynamics. We used two types of traps: (i) CDC Ondestepoort mobile light and (ii) unconventional type (hand-made). Traps were placed in different areas and localities in the counties of Timiş, Arad and Caraş-Severin. They were followed over three consecutive nights in each location. In total 28 samples were taken.

In addition, abiotic parameters monitoring was performed (minimum temperature, maximum temperature, relative humidity and wind speed), evaluating their influence on *Culicoides *population dynamics. Data on atmospheric temperature (maximum and minimum temperature), wind speed and relative humidity were taken at each trap using an anemometer and an ambient thermo-hygrometer; for a more precise expression of data a conjunction with data taken from the website of the National Agency of Meteorology of the two months was made.

A total of 4534 Diptera specimens were captured during this research in Timiş, Arad and Caraş-Severin Counties, including three species having the role of potential vectors for bluetongue, namely *Culicoides obsoletus *409 (9.02%), *Culicoides pulicaris *239 (5.27%) and *Culicoides nubeculosus *183 (4.03%).

Based on the preliminary data obtained in Western Romania, it is considered that the population dynamics of *Culicoides *is influenced by abiotic factors such as maximum temperature, minimum temperature, average temperature, wind speed and relative humidity.

Of all the abiotic factors monitored, the average temperature, relative humidity and wind speed have a major role in the variability of the total number of *Culicoides*. Dynamics of *Culicoides *population is positively correlated with minimum temperature (11.5-16 °C).

The results obtained in previous studies conducted in this part of Romania show that the maximum temperature explained most variability in the total number of *Culicoides *and wind speed at least. Comparing to them, the results obtained in this study render that during May-June 2013 increased values of wind speed (1.8-2.5 m/s) and low relative humidity (60%) had a more negative influence on insect population dynamics, as compared with the maximum (30 °C) and average (15-16 ° C) temperatures.

